# Narcissism, the Experience of Pain, and Risky Decision Making

**DOI:** 10.3389/fpsyg.2020.01128

**Published:** 2020-05-27

**Authors:** Melissa T. Buelow, Amy B. Brunell

**Affiliations:** ^1^Department of Psychology, The Ohio State University, Newark, OH, United States; ^2^Department of Psychology, The Ohio State University, Mansfield, OH, United States

**Keywords:** risky decision making, Iowa Gambling Task, pain, grandiose narcissism, vulnerable narcissism

## Abstract

Personality characteristics and situational factors are known to influence performance on behavioral decision making tasks; however, variability exists in the relationship between narcissism and decision making. In addition, recent research suggests that the presence of acute pain can negatively affect decisions, and even the threat of pain can also cause changes in decision making. Narcissists are known to experience social pain differently than non-narcissists, but relatively little is known about how physical pain is experienced. The present study examined the influence of both pain and narcissism on risky decision making task performance. Participants (*n* = 248) completed assessments of the narcissistic admiration and rivalry concept as well as vulnerable narcissism. They were asked to complete a pain recall task before administration of the Balloon Analog Risk Task (BART), Columbia Card Task (CCT), Game of Dice Task (GDT), and Iowa Gambling Task (IGT). Although individuals who recalled a socially painful experience took less risks on the IGT across trials, no effect of narcissism was seen on any of the tasks. Recalling a physically or socially painful situation did not negatively affect decision making on the BART, CCT, or GDT. Results are discussed in the context of previous research on narcissism, pain, and cognitive task performance.

## Introduction

Individuals engage in risk-taking behaviors with potential negative consequences for health and well-being. Researchers often use behavioral tasks, such as the Iowa Gambling Task (IGT; Bechara et al., 1994) and Balloon Analog Risk Task (BART; Lejuez et al., 2002), to assess risk-taking behavior and risky decision making in lab-based settings. Recently, significant interest has grown in determining the predictors of risky decision making, including in the domains of cognitive function, personality, and situational factors. Two topics that are of particular recent interest as potential predictors of risky decision making are narcissism and pain.

### Narcissism

Narcissism is described here as a personality trait found in the overall population. It is a complex construct, entailing a collection of traits that seemingly contradict one another. Grandiose narcissism (GN) is characterized by high self-esteem and extraversion, whereas vulnerable narcissism (VN) is characterized by low self-esteem and negative emotionality (e.g., Miller and Campbell, 2008), including greater anxiety and depression (Brunell and Buelow, 2019). Although distinguishing between GN and VN is important, GN is not conceptualized as a homogenous variable. One model of narcissism, the Narcissistic Admiration and Rivalry Concept (Back et al., 2013), distinguishes between an agentic dimension, termed *narcissistic admiration*, and an antagonistic dimension, termed *narcissistic rivalry*. Narcissistic admiration includes striving for uniqueness and realizing grandiose fantasies, as well as charming behavior (Back, 2018). Thus, narcissistic admiration reflects a self-promotion strategy. However, when the desired outcomes of status, praise, and admiration are thwarted, the strategy of narcissistic self-defense in the form of narcissistic rivalry may be used instead. Thus, narcissistic rivalry encompasses behavior dynamics including striving for supremacy, devaluation of others, as well as hostile and insensitive behavior, especially following conflict such as rejection and criticism. It is possible these two components may lead to differing responses following a painful experience, with narcissistic admiration’s effects thwarted and narcissistic rivalry’s effects more prominent on subsequent behaviors. One such behavior that could occur after a painful experience is decision making.

Research into the effects of narcissism on decision making are quite varied and can depend on how narcissism is assessed. Despite evidence that narcissists take greater risks in leadership positions (Chatterjee and Hambrick, 2007), in finance (Foster et al., 2009a, b), and across risk-taking behaviors (Buelow and Brunell, 2018), data is mixed when behavioral tasks are examined. Grandiose narcissists take greater risks on a monetary gambling task (Yang et al., 2018b) but not on tasks such as the BART or IGT (Crysel et al., 2013; Brunell and Buelow, 2017). Specific narcissistic traits such as entitlement, rather than GN, do relate to performance on behavioral tasks (Brunell and Buelow, 2017), indicating the need for a more detailed examination of the elements of narcissism that affect risk-taking behavior. Finally, situational factors, such as social support, can also affect a narcissist’s performance on behavioral tasks (e.g., Carre and Jones, 2016; Yang et al., 2018a). Thus, the current literature is mixed with regard to narcissism’s effects on behavioral tasks. In addition, to the best of our knowledge, no research to date examined this topic from the lens of the Narcissistic Admiration and Rivalry Concept (Back et al., 2013), a conceptualization that appears well-designed to assess narcissism’s effects on decision making following a painful experience.

### Pain

Pain can be acute or chronic and both types of pain can negatively affect attention, in turn distracting valuable resources from the task at hand (Van Damme et al., 2002). Pain can negatively affect attention and working memory (e.g., Crombez et al., 1996, 1998; Dick and Rashiq, 2007; Dohrenbusch et al., 2008; Moore et al., 2012), both of which are necessary to complete many behavioral risky decision making tasks. Acute (Porcelli and Delgado, 2009; Koppel et al., 2017; Barnhart et al., 2019) and chronic (Apkarian et al., 2004; Tamburin et al., 2014; Muñoz Ladrón de Guevara et al., 2018) pain impair performance on the IGT and other tasks, in that participants are riskier in their decisions when in pain versus no pain. One theory behind riskier decision making during a painful experience is that participants are using the positive “win” element of making a riskier decision to offset the negative experience of pain (Koppel et al., 2017). Even the threat of additional pain in the future can negatively affect performance on tasks such as the IGT and BART (e.g., Barnhart et al., 2019).

The negative effects of pain on decision making and other cognitive processes are not limited to just physical pain. Social pain activates similar brain structures as physical pain (e.g., anterior cingulate cortex; Eisenberger et al., 2003), meaning that experiencing social pain can be just as painful as experiencing physical pain. Social pain most often occurs when an individual is ostracized – is excluded and ignored. Previous research suggests that experiencing social pain can increase risky decision making (Duclos et al., 2013; Buelow et al., 2015; Buelow and Wirth, 2017). In addition, participants asked to recall a burdensome friend reported greater levels of physical pain and negative affect (Okdie and Wirth, 2018), indicating that even recalling a previous experience of social pain can negatively affect the individual in the present moment.

### The Present Study

The present study sought to examine the influence of narcissism and pain on risky decision making. GN is associated with a hypersensitivity to social exclusion and threatening situations (e.g., Kelsey et al., 2001; Sommer et al., 2009), and shows increased activation in neural pain areas during such experiences (Cascio et al., 2015). In addition, narcissists do experience physical pain, reporting worse mood following a cold pressor task (Brunell et al., 2020). In the present study, we had participants self-report a time in which they experienced social or physical pain, then completed risky decision making tasks. Several hypotheses were examined. Experiencing social or physical pain can lead to impaired performance on cognitive tasks, including on those that assess decision making. To our knowledge, no research has specifically examined how recalling a previously painful experience might in turn affect cognitive abilities in the present moment. We hypothesized recalling a painful experience would result in riskier decisions than in the control condition, consistent with previous research indicating experiencing an acute pain affects decisions. It is also possible that following a painful experience, or in this case recalling a painful experience, an individual with high levels of narcissistic admiration and rivalry may be inclined to take risks in order to regain their grandiose sense of self. Thus, we predicted higher levels of narcissistic admiration and rivalry would predict greater risky decision making following a pain recall task (no specific hypothesis was made about VN).

## Materials and Methods

### Participants

Participants were 248 undergraduate students at a regional campus of a large University (79 males, *M_age_* = 18.62, *SD*_*age*_ = 2.03). Most self-identified as Caucasian or African-American (see Table 1). All were enrolled in psychology courses in which course credit was provided for participation in research studies. Participants were not paid for their participation, nor were real incentives tied to task performance.

**TABLE 1:** Demographic information and study variable means and standard deviations.

	**Recall condition**			
**Variable**	**Control**	**Anger**	**Social pain**	**Physical pain**
*n*	114	45	44	45
Gender	36 Males	15 Males	12 Males	16 Males
Age	18.38 (0.74)	19.07 (4.05)	18.49 (1.08)	18.89 (1.91)
Ethnicity	62.5%	73.3%	58.1%	59.1%
	Caucasian	Caucasian	Caucasian	Caucasian
NARQ-A	2.91 (0.83)	3.14 (1.08)	3.03 (1.06)	3.24 (1.14)
NARQ-R	1.93 (0.73)	1.90 (0.75)	2.12 (1.00)	1.89 (0.97)
HSNS	2.70 (0.71)	2.65 (0.77)	2.70 (0.65)	2.53 (0.70)
PANAS-P	2.70 (0.91)	2.50 (0.83)	2.59 (0.88)	2.58 (0.97)
PANAS-N	1.43 (0.50)	1.74 (0.75)	1.54 (0.48)	1.47 (0.44)
BART	23.49 (13.17)	25.01 (14.39)	20.77 (14.13)	25.72 (11.98)
CCT	13.07 (4.71)	12.62 (5.27)	14.47 (4.80)	13.80 (5.35)
GDT	4.74 (9.29)	0.59 (10.14)	4.43 (11.43)	0.85 (12.09)
IGT 1-40	−2.97(9.21)	−1.45(12.13)	−2.60(9.97)	−2.55(12.14)
IGT 41-100	−6.29(18.45)	1.59 (23.29)	1.72 (24.37)	−3.98(17.27)

*NARQ, Narcissistic Admiration and Rivalry Questionnaire; HSNS, Hypersensitive Narcissism Scale; PANAS, Positive and Negative Affect Schedule; BART, Balloon Analog Risk Task, average adjusted pumps per balloon; GDT, Game of Dice Task, net score; CCT, Columbia Card Task, average selections; IGT, Iowa Gambling Task, advantageous minus disadvantageous selections during early (1–40) and later (41–100) trials.*

### Narcissism Measures

The Narcissistic Admiration and Rivalry Questionnaire (NARQ; Back et al., 2013) assesses these characteristics of GN by having participants respond to a series of 18 items using a 1 (*not agree at all*) to 6 (*agree completely*) scale. Admiration items focus on self-enhancement (e.g., being famous, special, and great) whereas rivalry items focus on self-defense (e.g., enjoying failure of rivals, annoyance at criticism). Higher average scores indicate greater narcissistic admiration (*M* = 3.04, *SD* = 0.99, α = 0.86) and rivalry (*M* = 1.96, *SD* = 0.84, α = 0.86).

The Hypersensitive Narcissism Scale (HSNS; Hendin and Cheek, 1997) assesses VN. Participants respond to a series of 10 items using a 1 (*very uncharacteristic*) to 5 (*very characteristic*) scale (e.g., dislike sharing credit with others and interpreting remarks in a personal way). Higher average scores indicate greater VN (*M* = 2.66, *SD* = 0.70, α = 0.75).

### Risky Decision Making Measures

On the BART (Lejuez et al., 2002), participants pump up a series of 30 balloons, earning five cents per pump. Participants can pump up the balloon as much as they want, clicking “collect $$$” to bank that money. If the balloon pops, however, the money earned on that balloon is lost. Unknown to participants, each balloon has a breaking point between 1 and 128 pumps. To earn money, participants must stop pumping the balloon before it pops and bank the earned money. Average adjusted number of pumps per balloon was calculated, with higher scores indicating greater risky decision making.

The Columbia Card Task (CCT; Figner et al., 2009) assesses risky decision making by having participants turn over a set of 32 cards. Some are “win” cards, earning 10 or 30 points, and some are “loss” cards (1 or 3), subtracting 250 or 750 points. At the start of each trial, participants are told the win points, loss points, and number of loss cards. The “cold” version was administered, which had participants indicate the total number of cards to turn over. No feedback about their selection is provided before the next trial begins. The average number of cards per trial was calculated, with higher scores indicative of riskier decision making.

The Game of Dice Task (GDT; Brand et al., 2007) assesses risky decision making by having participants guess the roll of a die. Participants place a bet on a series of 1, 2, 3, or 4 digits that matching their prediction. If the prediction is correct, they win money and if it is wrong they lose money. Participants are told the relative risk associated with each decision. A total score was calculated by subtracting the number of disadvantageous bets (1, 2) from the number of advantageous bets (3, 4), with lower scores indicative of riskier decision making.

The IGT (Bechara et al., 1994; Bechara, 2007) was created to assess real-world decision making impairments among individuals with frontal lobe injuries. Participants are given $2,000 and told to maximize profit over a series of 100 selections from one of four decks (A, B, C, and D). On each trial, participants win some money but might also lose some money. Selecting Deck A or B results in larger immediate rewards, but the losses outweigh the gains (long-term negative consequences). Selecting Deck C or D results in smaller immediate rewards, but the gains outweigh the losses (long-term positive consequences). Performance is divided into decisions made under ambiguity, when not much is known about the decks (Trials 1–40), and decisions made under risk, when participants are at least somewhat aware of the relative risks and benefits of each deck (Trials 41–100; Brand et al., 2007). For the present study, performance was examined by subtracting the disadvantageous selections (A + B) from the advantageous selections (C + D) during the early (1–40) and later (41–100) trials.

Although these four tasks all assess risky decision making, each differs from the others in some way. The IGT assesses elements of both decision making under ambiguity and decision making under risk, given that participants do not know much about the pros and cons of each deck during early decisions compared to later decisions. Decision making under ambiguity turns into decision making under risk as participants utilize the feedback from each decision to change their perception of each deck and thus their decision making strategy. On the CCT, participants do not receive feedback on the outcomes of their decisions, but start the task with a wealth of information to determine how risky a decision actually is. Participants can balance the knowledge about the number of loss cards and their value with the value of the gain cards to arrive at an optimal decision. On the GDT, participants are also given explicit information about the wins/losses associated with each decision, but it is up to the participants to determine the probability associated with winning/losing on a particular trial. The BART introduces an element of randomness, as the explosion point varies across trials and can lead to a different decision making strategy.

### Procedure

The University’s Institutional Review Board approved the study and all participants provided informed consent. Participants were told that they were part of a study examining predictors of cognitive task performance. They then completed the NARQ, HSNS, and demographic questionnaire in a random order. Next, participants were assigned to one of four recall conditions: (1) previous social pain (*n* = 44; recall a time they felt socially ostracized); (2) previous physical pain (*n* = 45; recall a time they felt a high level of physical pain); (3) previous anger [*n* = 45; recall a time they experienced a high level of anger (emotional control condition)]; or (4) control (*n* = 114; write about activities completed earlier in the day). Participants were not given further direction as to what the recalled situation should entail, thus they might have written about ostracism by a familiar person or ostracism by an unknown or casual acquaintance. They then completed the Positive and Negative Affect Schedule (Watson et al., 1988) to assess their post-manipulation level of positive and negative affect before completing the BART, CCT, GDT, and IGT in a counterbalanced order. Participants were then debriefed and course credit assigned. Of note, the control condition was oversampled to allow for analyses that collapsed across the recall conditions (*n* = 134 across the three recall conditions).

### Data Analysis and Results

Due to computer malfunction, data was missing for the BART (*n* = 9), CCT (*n* = 11), GDT (*n* = 16), and IGT (*n* = 10) (3.6–6.5% of data by task). Demographic information and variable means and standard deviations are presented in Table 1. Gender was correlated with performance on the IGT, so gender was included as a covariate in the remaining analyses.

First, hypotheses regarding the influence of recalled pain on decision making were examined. To assess whether the pain recall tasks elicited an emotional response, a series of ANOVAs were conducted on the PANAS positive and negative subscales, with gender as a covariate. There was not a significant difference in positive affect across pain recall conditions, *F*(3, 240) = 0.74, *p* = 0.529, η^2^ = 0.01; however, there was a significant between-groups difference in negative affect, *F*(3, 240) = 3.93, *p* = 0.009, η^2^ = 0.05. The anger recall condition reported greater negative affect than the control (*p* = 0.001), physical pain (*p* = 0.018), and social pain (*p* = 0.030) conditions. No differences emerged between the remaining groups (*p*s > 0.477).

**FIGURE 1:**
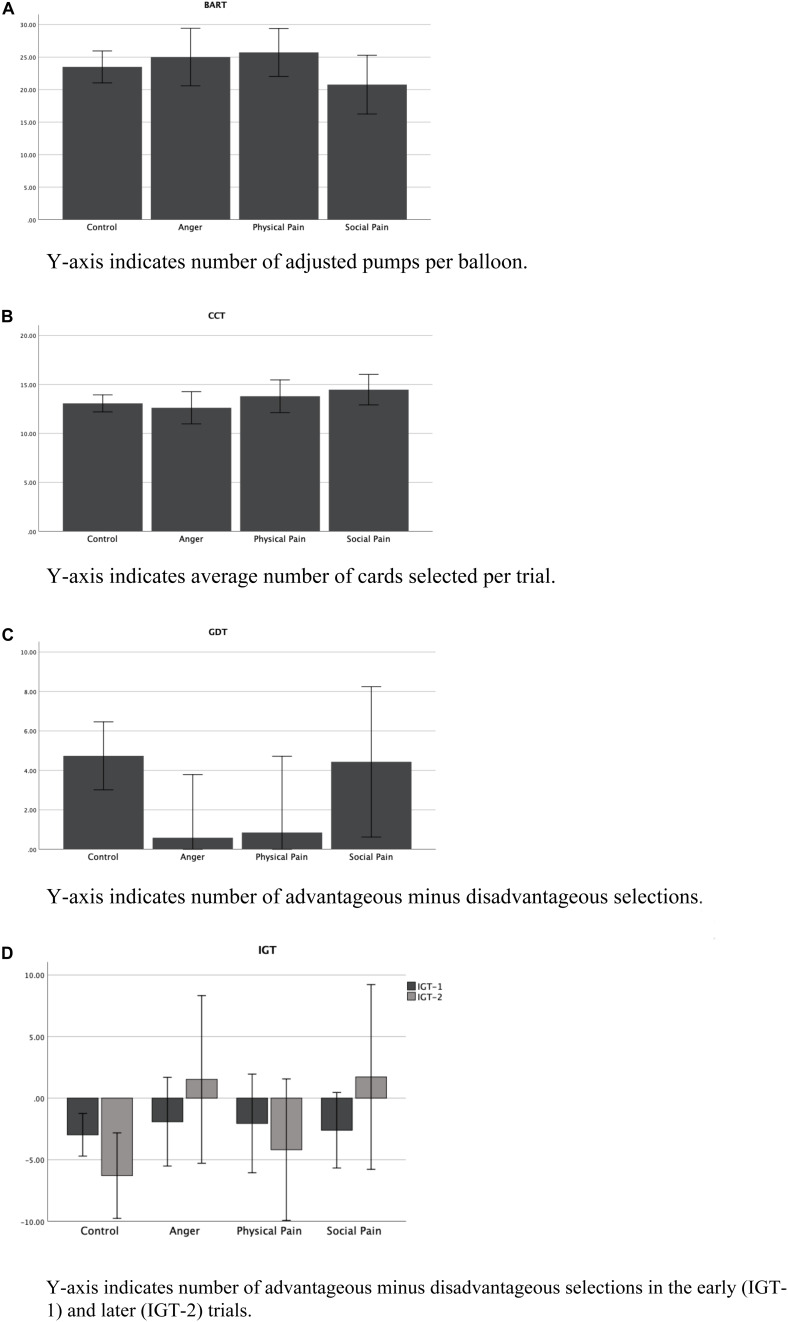
Performance on the BART **(A)**, CCT **(B)**, GDT **(C)**, and IGT **(D)** as a function of pain recall condition.

A series of ANOVAs were conducted on the BART, CCT, and GDT, including gender as a covariate. Because the IGT is separated into two blocks of trials, a mixed ANOVA was also conducted with trial block (1–40 and 41–100) as the repeated-measures factor and pain recall condition (social, physical, anger/emotional control, and control) as the between-subjects factor. Gender was again entered as a covariate. No significant pain-recall group effects were seen on the BART, *F*(3, 235) = 0.79, *p* = 0.502, η^2^ = 0.010; CCT, *F*(3, 233) = 1.62, *p* = 0.185, η^2^ = 0.021; or GDT, *F*(3, 228) = 2.62, *p* = 0.052, η^2^ = 0.034. In addition, gender was not associated with performance on any of these tasks (*p*s > 0.128). For the IGT, the main effects of Block, *F*(1, 229) = 2.00, *p* = 0.158, η^2^ = 0.009, and Group, *F*(3, 229) = 2.12, *p* = 0.098, η^2^ = 0.027, were not significant. Men showed more advantageous decisions than women, *F*(1, 229) = 5.85, *p* = 0.016, η^2^ = 0.025, but there was not an interaction with Block, *F*(1, 229) = 1.54, *p* = 0.215, η^2^ = 0.007. There was a significant Block × Group interaction, *F*(3, 229) = 3.55, *p* = 0.015, η^2^ = 0.044. Among participants in the control group, less risky decisions were seen in the earlier than in the later trials, *p* = 0.034, whereas those in the social pain recall group made less risky decisions in the later than in the earlier trials, *p* = 0.036 (see Figure 1).

**FIGURE 2:**
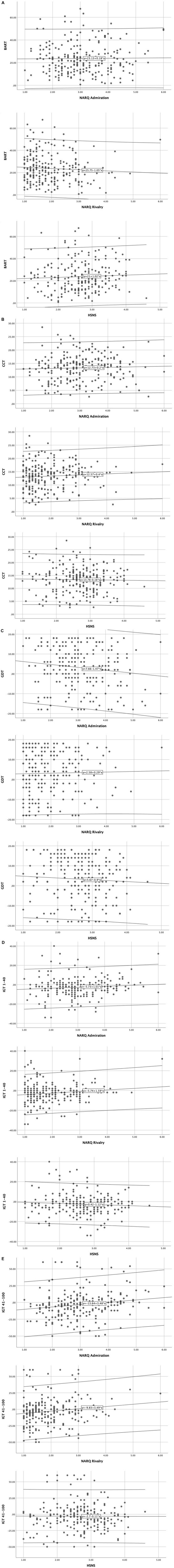
Relationship between performance on the BART **(A)**, CCT **(B)**, GDT **(C)**, IGT-1 **(D)**, and IGT-2 **(E)**, and the narcissism measures [admiration (1), rivalry (2), and vulnerable (3)].

Next, hypotheses regarding narcissism’s effects on decision making were examined via a series of linear regressions. The pain recall conditions and centered narcissism variables were entered in Step 1. The interaction between the narcissism variables and pain recall conditions were entered in Step 2. As recall condition was a categorical variable, the conditions were first dummy-coded as follows: Social pain (1 = Social pain and 0 = all other conditions), Physical pain (1 = Physical pain and 0 = all other conditions), and Anger (1 = Anger and 0 = all other conditions) (see Figure 2).

Results of the regression analyses are presented in Table 2. To account for multiple comparisons, the Bonferroni correction was applied. Results are considered significant at the *p* < 0.007 level (0.05/7 Step 2 predictors per regression). No significant associations emerged for performance on the BART, CCT, or IGT-1-40. No significant associations emerged at the *p* < 0.007 level for the GDT (NARQ-A was significant at *p* = 0.02 level) or the IGT-41-100 (conditions were significant at the *p* = 0.03 level).

**TABLE 2:** Results of regression analyses on decision making and narcissism.

**Variable**	***F***	***p***	***R*^2^**	**β**	***t***	***p***	**VIF**
**BART**							
**Step 1: Narcissism, condition**	0.97	0.446	0.03				
Social pain				–0.08	–1.13	0.259	1.15
Physical pain				0.06	0.78	0.435	1.17
Anger				0.01	0.08	0.938	1.15
NARQ-A				0.06	0.79	0.432	1.34
NARQ-R				–0.11	–1.38	0.170	1.45
HSNS				0.09	1.31	0.192	1.12
**Step 2: Narcissism × Condition**	0.67	0.811	0.04				
Social pain				–0.07	–0.98	0.326	1.19
Physical pain				0.05	0.71	0.479	1.22
Anger				0.01	0.18	0.856	1.18
NARQ-A				–0.08	–0.59	0.558	3.99
NARQ-R				–0.15	–1.15	0.251	3.94
HSNS				0.09	0.91	0.366	2.31
NARQ-A × Social pain				0.07	0.67	0.507	2.38
NARQ-A × Physical pain				0.15	1.46	0.145	2.39
NARQ-A × Anger				0.06	0.61	0.541	2.07
NARQ-R × Social pain				0.02	0.15	0.879	1.94
NARQ-R × Physical pain				0.05	0.49	0.622	2.44
NARQ-R × Anger				0.03	0.37	0.715	2.84
HSNS × Social pain				–0.02	–0.26	0.795	1.62
HSNS × Physical pain				–0.00	–0.03	0.979	1.57
HSNS × Anger				0.00	0.05	0.964	1.76

**CCT**							
**Step 1: Narcissism, condition**	0.82	0.555	0.02				
Social pain				0.10	1.43	0.155	1.14
Physical pain				0.06	0.78	0.438	1.17
Anger				–0.03	–0.48	0.634	1.15
NARQ-A				0.00	0.01	0.990	1.35
NARQ-R				0.07	0.92	0.357	1.46
HSNS				–0.05	–0.70	0.487	1.12
**Step 2: Narcissism × Condition**	0.76	0.722	0.05				
Social pain				0.12	1.61	0.110	1.18
Physical pain				0.05	0.68	0.500	1.24
Anger				–0.03	–0.45	0.653	1.16
NARQ-A				–0.05	–0.39	0.697	3.92
NARQ-R				0.11	0.87	0.386	3.93
HSNS				–0.11	–1.06	0.290	2.30
NARQ-A × Social pain				–0.06	–0.62	0.537	2.36
NARQ-A × Physical pain				0.12	1.17	0.244	2.40
NARQ-A × Anger				0.02	0.25	0.800	2.02
NARQ-R × Social pain				–0.06	–0.53	0.597	1.95
NARQ-R × Physical pain				–0.04	–0.35	0.726	2.45
NARQ-R × Anger				0.05	0.49	0.627	2.81
HSNS × Social pain				–0.01	–0.09	0.931	1.62
HSNS × Physical pain				0.08	0.97	0.336	1.56
HSNS × Anger				0.02	0.24	0.811	1.76

**GDT**							
**Step 1: Narcissism, condition**	2.31	0.035	0.06				
Social pain				0.00	0.00	1.000	1.14
Physical pain				–0.11	–1.59	0.113	1.17
Anger				–0.13	–1.84	0.068	1.14
NARQ-A				–0.18	–2.35	0.020	1.37
NARQ-R				0.13	1.63	0.104	1.46
HSNS				–0.10	–1.47	0.144	1.11
**Step 2: Narcissism × Condition**	1.27	0.226	0.08				
Social pain				–0.01	–0.09	0.926	1.17
Physical pain				–0.10	–1.41	0.159	1.24
Anger				–0.13	–1.89	0.061	1.16
NARQ-A				–0.06	–0.48	0.635	3.89
NARQ-R				–0.04	–0.34	0.734	3.88
HSNS				–0.03	–0.26	0.794	2.21
NARQ-A × Social pain				–0.11	–1.13	0.258	2.33
NARQ-A × Physical pain				–0.11	–1.12	0.265	2.36
NARQ-A × Anger				–0.04	–0.42	0.677	2.08
NARQ-R × Social pain				0.16	1.41	0.161	1.96
NARQ-R × Physical pain				0.20	1.99	0.048	2.38
NARQ-R × Anger				0.03	0.29	0.772	2.82
HSNS × Social pain				–0.08	–0.96	0.340	1.46
HSNS × Physical pain				–0.07	–0.87	0.384	1.52
HSNS × Anger				–0.03	–0.40	0.691	1.72

**IGT 1-40**							
**Step 1: Narcissism, condition**	1.30	0.256	0.03				
Social pain				0.01	0.18	0.856	1.15
Physical pain				0.02	0.30	0.767	1.18
Anger				0.08	1.14	0.256	1.15
NARQ-A				0.02	0.28	0.783	1.37
NARQ-R				0.15	1.85	0.066	1.46
HSNS				–0.11	–1.53	0.128	1.10
**Step 2: Narcissism × Condition**	1.63	0.067	0.10				
Social pain				0.01	0.14	0.886	1.19
Physical pain				0.01	0.11	0.914	1.27
Anger				0.08	1.09	0.276	1.17
NARQ-A				–0.08	–0.60	0.548	4.26
NARQ-R				0.19	1.44	0.150	3.95
HSNS				–0.05	–0.47	0.636	2.32
NARQ-A × Social pain				–0.09	–0.89	0.374	2.53
NARQ-A × Physical pain				0.21	2.04	−0.043	2.47
NARQ-A × Anger				0.05	0.49	0.625	2.17
NARQ-R × Social pain				0.06	0.51	0.610	1.97
NARQ-R × Physical pain				0.03	0.31	0.759	2.30
NARQ-R × Anger				–0.13	–1.48	0.142	2.94
HSNS × Social pain				–0.14	–1.64	0.102	1.63
HSNS × Physical pain				–0.02	–0.31	0.759	1.46
HSNS × Anger				–0.00	–0.04	0.966	1.69

**IGT 41-100**														
**Step 1: Narcissism, condition**	2.60	0.019	0.07				
Social pain				0.15	2.13	0.034	1.15
Physical pain				0.04	0.53	0.598	1.18
Anger				0.16	2.26	0.025	1.15
NARQ-A				0.12	1.58	0.115	1.37
NARQ-R				0.08	1.03	0.305	1.46
HSNS				–0.02	–0.34	0.736	1.10
**Step 2: Narcissism × Condition**	1.22	0.258	0.08				
Social pain				0.15	2.06	0.041	1.19
Physical pain				0.02	0.27	0.787	1.27
Anger				0.16	2.23	0.027	1.17
NARQ-A				0.01	0.07	0.948	4.26
NARQ-R				0.13	0.99	0.324	3.95
HSNS				0.00	0.01	0.996	2.32
NARQ-A × Social pain				0.03	0.33	0.742	2.53
NARQ-A × Physical pain				0.14	1.32	0.189	2.47
NARQ-A × Anger				0.05	0.49	0.623	2.17
NARQ-R × Social pain				0.03	0.27	0.785	1.97
NARQ-R × Physical pain				–0.07	–0.67	0.506	2.30
NARQ-R × Anger				–0.06	–0.68	0.499	2.94
HSNS × Social pain				–0.06	–0.74	0.460	1.63
HSNS × Physical pain				–0.03	–0.32	0.751	1.46
HSNS × Anger				0.01	0.14	0.888	1.69

*NARQ, Narcissistic Admiration and Rivalry Questionnaire; HSNS, Hypersensitive Narcissism Scale; BART, Balloon Analog Risk Task, average adjusted pumps per balloon; GDT, Game of Dice Task, net score; CCT, Columbia Card Task, average selections; IGT, Iowa Gambling Task, advantageous minus disadvantageous selections during early (1–40) and later (41–100) trials.*

## Discussion

The present study examined the effects of both a pain-recall manipulation and the personality characteristic of narcissism on decision making. We hypothesized that recalling a painful experience would result in riskier decisions, as acute pain (Porcelli and Delgado, 2009; Koppel et al., 2017; Barnhart et al., 2019) and recalling a previously painful experience (Okdie and Wirth, 2018) can negatively affect individuals. Contrary to prediction, minimal recall manipulation effects were seen across the decision making tasks. The only significant findings were on the IGT, as participants who recalled a socially painful experience were less risky as the task progressed whereas the control participants instead became riskier as the task progressed. Although unexpected, poor performance among healthy controls (and those in control conditions) is commonly found on the IGT (e.g., Steingroever et al., 2013). It is possible, however, that the unusually low performance of the control group – and the increased preference for disadvantageous decks as the task progressed – affected our ability to detect group differences in the physical pain recall condition and on other tasks.

Our finding of improved performance on the IGT as a function of social pain recall condition generally runs counter to prediction and previous research suggesting pain impairs decision making. It is possible that our pain recall manipulations tapped into individuals’ current mood states, as previous research found both state positive and negative mood can affect decision making (e.g., Cryder et al., 2008; de Vries et al., 2008; Buelow et al., 2013). Those in a more negative mood following the recall might attempt to repair mood by “winning” on the various tasks, leading to a strategy of focusing on the feedback to learn to decide advantageously. However, examination of the PANAS post-manipulation indicated only the anger recall condition led to a significant difference in positive or negative affect. Additional research is needed to both replicate this finding and determine what might be causing a post-pain recall improvement in decision making on the IGT but not the GDT. Our effect sizes for the ANOVAs fell in the small range and it is possible that our smaller sample size might have hampered our ability to detect significant small effects between pain recall groups.

We also predicted relationships between narcissistic admiration and rivalry (GN) and risky decision making (no prediction was made for VN). Specifically, we hypothesized higher GN would be associated with riskier decisions than lower levels of these characteristics. No support was found for this hypothesis. No narcissism variables were significantly related to decision making task scores at the *p* < 0.01 level. The narcissism and pain recall condition interactions all fell in the small to moderate range of effect sizes, and our smaller sample size may have also hindered our ability to detect significant but small effects. However, our limited findings are consistent with previous research showing both the assessment of narcissism and the behavioral task utilized can lead to varied relationships between narcissism and risky decision making (e.g., Crysel et al., 2013; Carre and Jones, 2016; Brunell and Buelow, 2017; Yang et al., 2018a, b). Some tasks rely more on explicit (GDT) versus implicit (IGT) information about the relative risks and benefits associated with each decision, leading to differing levels of effort and attention required to learn the optimal strategy. Still other tasks might better resemble real-world games (BART and GDT), leading participants to use previously learned information (that may or may not apply to the lab task) when making decisions. It is also possible that narcissism’s effects on decision making and risk-taking behavior occur when there is a potential for others to learn about the outcomes, such as can occur in real-world decision making settings. Future research could examine how decisions change as a function of narcissists deciding individually versus in a group setting.

Several limitations exist which may have affected the results. As previously stated, the control group significantly preferred the disadvantageous decks, contrary to the IGT creator’s intention but potentially reflecting the prominent Deck B phenomenon seen across studies (e.g., Lin et al., 2007). Although we assessed changes in positive and negative mood after the pain recall task, we did not include a measure of current pain experience. Our sample, though diverse, was comprised of undergraduate student participants and may not reflect decision making tendencies of non-student participants. We did not tie real financial incentives to participation in the study nor to performance on the tasks. Although this lack of financial incentives is common in psychological studies utilizing the BART, CCT, GDT, and IGT, research in economics points toward real incentives influencing and even improving decision making (e.g., Hertwig and Ortmann, 2001; Cohn et al., 2015; but see Scheres et al., 2010). Emotions can negatively affect decision making (e.g., Engelmann and Hare, 2018), but factors such as the type of behavioral task, how negative or positive affect are induced, and the strength of the mood induction method, in addition to the use of monetary payments, can affect this process. Our small sample size could negatively affect our power to detect small effects. In addition, we experienced a loss of data on several of the decision making tasks, which negatively affected our sample sizes for each analysis. It is possible the results would turn out differently if a different measure of narcissism was utilized, such as the Narcissistic Personality Inventory (NPI). It is also possible that narcissists are biased in their recall about themselves (consistent with Jones and Brunell, 2014), in that they strive to see themselves in positive agentic ways that are lacking in complexity and are self-aggrandizing (e.g., Rhodewalt and Morf, 1995). These biases may also limit how much self-related information they can store about themselves (e.g., Fukushima and Hosoe, 2011), which could negatively affect a recall task such as was used in the present study. It is also possible that the individual referenced in the socially painful experience was particularly impactful in that participants may have experienced ostracism by a familiar person as worse than ostracism by an unknown or casual acquaintance. Use of an experimental manipulation of pain, such as Cyberball (social pain; Williams et al., 2000) and a cold pressor (physical pain), could offset some of these concerns. Future research should attempt to determine when narcissists take risks and why, as well as how the experience or avoidance of pain might affect this process. In addition, future research should begin to examine how performance on decision making tasks changes on each successive selection, and whether narcissists may show decision making patterns in behavioral models that do not appear when the standard scoring approaches are utilized.

## Data Availability Statement

The datasets generated for this study are available on request to the corresponding author.

## Ethics Statement

The studies involving human participants were reviewed and approved by The Ohio State University Institutional Review Board. Written informed consent for participation was not required for this study in accordance with the national legislation and the institutional requirements. Participants provided verbal consent.

## Author Contributions

MB designed the study, collected and analyzed data, and drafted the manuscript. AB designed the study and revised the manuscript.

## Conflict of Interest

The authors declare that the research was conducted in the absence of any commercial or financial relationships that could be construed as a potential conflict of interest.
